# Evaluating the Feasibility and Acceptability of a Digital Pre-Exposure Prophylaxis Navigation and Activation Intervention for Racially and Ethnically Diverse Sexual and Gender Minority Youth (PrEPresent): Protocol for a Pilot Randomized Controlled Trial

**DOI:** 10.2196/50866

**Published:** 2023-09-29

**Authors:** Jacob B Stocks, Sam Calvetti, Matthew T Rosso, Lindsay Slay, Michele Kipke, Manuel Puentes, Lisa B Hightow-Weidman

**Affiliations:** 1 Institute on Digital Health and Innovation College of Nursing Florida State University Tallahassee, FL United States; 2 Division of Research on Children, Youth, and Families Department of Pediatrics Children's Hospital Los Angeles Los Angeles, CA United States; 3 Department of Population and Public Health Sciences Keck School of Medicine University of Southern California Los Angeles, CA United States

**Keywords:** PrEP, mobile health, LGBTQ, transgender, adolescent, mobile phone

## Abstract

**Background:**

To end the HIV epidemic by 2030, we must double down on efforts to tailor prevention interventions to both young men who have sex with men and transgender and nonbinary youth. There is an urgent need for interventions that specifically focus on pre-exposure prophylaxis (PrEP) uptake in sexual and gender minority youth (SGMY) populations. There are several factors that impact the ability of SGMY to successfully engage in the HIV prevention continuum, including uptake of PrEP. Patient activation, having the knowledge, skills, and self-efficacy to manage one’s health, is an important indicator of willingness and ability to manage one’s own health and care autonomously. Patient navigation also plays an important role in helping SGMY access PrEP and PrEP care, as navigators help guide patients through the health care system, set up medical appointments, and get financial, legal, and social support.

**Objective:**

This study aims to evaluate the feasibility and acceptability of a digital PrEP navigation and activation intervention among a racially and ethnically diverse sample of SGMY living in the Los Angeles area.

**Methods:**

In phase 1, we will conduct formative research to inform the development of PrEPresent using qualitative data from key informant interviews involving PrEP care providers and navigators and working groups with SGMY. In phase 2, we will complete 2 rounds of usability testing of PrEPresent with 8-10 SGMY assessing both the intervention content and mobile health delivery platform to ensure features are usable and content is understood. In phase 3, we will conduct a pilot randomized controlled trial to evaluate the feasibility and acceptability of PrEPresent. We will randomize, 1:1, a racially and ethnically diverse sample of 150 SGMY aged 16-26 years living in the Los Angeles area and follow participants for 6 months.

**Results:**

Phase 1 (formative work) was completed in April 2021. Usability testing was completed in December 2021. As of June 2023, 148 participants have been enrolled into the PrEPresent pilot randomized controlled trial (phase 3). Enrollment is expected to be completed in July 2023, with final results anticipated in December 2023.

**Conclusions:**

The PrEPresent intervention aims to bridge the gaps in PrEP eligibility and PrEP uptake among racially and ethnically diverse SGMY. By facilitating the delivery of PrEP navigation and focusing on improving patient activation, the PrEPresent intervention has the potential to positively impact the PrEP uptake cascade in the HIV care continuum as well as serve as a model for the tailoring of PrEP interventions based on behavior-based qualifications for PrEP instead of generalized gender-based eligibility.

**Trial Registration:**

ClinicalTrials.gov NCT05281393; https://clinicaltrials.gov/ct2/show/NCT05281393

**International Registered Report Identifier (IRRID):**

DERR1-10.2196/50866

## Introduction

### Background

Because young men who have sex with men (YMSM) account for the vast majority of new HIV infections each year, HIV-prevention research has prioritized this population [1,2]. Among Black men who have sex with men newly diagnosed with HIV in 2017, 34% were between the ages of 13 and 24 years and 41% were between the ages of 25 and 35 years [3]. Rates of new infections among Latino YMSM are similarly alarming—while overall rates of HIV infection among this population declined from 2005 to 2014, new infections among 13-24 year old Latino YMSM increased by 87% [4]. Less is known about the engagement of transgender, nonbinary, and other gender nonconforming youth within the HIV care continuum.

Transgender and nonbinary identities are growing, especially among youth, at a fast rate. In a 2022 US Centers for Disease Control and Prevention (CDC) summary, it was estimated that over 1.6 million members of the US population identify as transgender people (0.5% of the population), and about 300,000 youth (1.4%) identify as transgender people [5]. Of these estimates, 38.5% are transgender women, 35.9% are transgender men, and 25.6% are gender nonconforming. In a 2021 lesbian, gay, bisexual, transgender, and queer (LGBTQ) sampling study of the United States, it was estimated that about 11% of LGBTQ adults identify as nonbinary. This percentage was approximated to just over 1 million nonbinary people (aged 18-60 years) living in the United States at the time of the report [6].

Emerging studies are examining the HIV incidence among transgender and nonbinary people. In a 2019 cohort study of 1608 transgender women, 42% of participants tested positive for HIV. In total, 82% of participants reported they had been tested in the last 12 months, and 96% reported being tested in their lifetime [7]. According to a 2018 analysis of care among transgender men living with HIV from 2009 to 2014, 69% of respondents had undetectable viral loads but only 40% reported HIV or sexually transmitted infection (STI) prevention counseling by a health care professional [8]. In a recent report of new HIV infections among transgender people, sexual contact exposure accounted for 90% of infections in transgender women, 73% of transgender men, and 82% of infections within an “additional gender identity” category [9], highlighting the importance of extending HIV-related interventions to all individuals based on their risk factors for HIV transmission as opposed to gender identity.

If we are to end the HIV epidemic by 2030, we must double down on efforts to tailor prevention interventions to both YMSM and transgender and nonbinary youth, hereafter referred to as sexual and gender minority youth (SGMY), especially youth of color. Pre-exposure prophylaxis (PrEP) uptake has remained exceedingly low among SGMY [10-12]. While there is now substantial evidence demonstrating that YMSM have high levels of PrEP awareness [13,14], very few have ever used PrEP. Strauss et al [14] found that 68% of YMSM recruited from 3 cities (Atlanta, Chicago, and New York City) reported awareness of PrEP; however, only 9% reported having ever used PrEP. The Healthy Young Men’s Cohort Study of Black and Latino YMSM has shown that while 90% of YMSM are aware of PrEP and 86% are eligible for PrEP (using CDC criteria), only 23% have ever been prescribed PrEP and just 8% report currently using PrEP [15]. In a 2017 national digital study of transmasculine individuals, 24.2% of participants met criteria for PrEP eligibility based on behavior in the last 6 months. Of those eligible, only 64.9% had received a HIV test in the last year, 33.9% reviewed PrEP information from their provider, and only 10.9% were currently taking PrEP [16]. Within the Transgender Youth of Color Cohort Study, 67% of nonbinary youth assigned female at birth and 65% of transgender men reported condomless sexual interactions in the last 6 months. Despite these rates of condomless sex within the cohort, only 8% had taken PrEP in their lifetime and 3% of participants reported actively taking PrEP [17]. This underscores the urgent need for interventions that specifically focus on PrEP uptake within SGMY populations.

There are several factors that impact one’s ability to successfully engage in the HIV prevention continuum, including uptake of PrEP. For example, individual factors such as illicit drug use and depression have been found to create disparities to accessing HIV prevention services [18-20]. In addition, sociocultural and structural determinants, such as inadequate access to health care, mistrust of health care providers, food insecurity, and residential instability, have also been found to serve as barriers to both HIV prevention and PrEP uptake [13,21,22].

In one study of young sexual minority men (n=492), only 14% reported having ever used PrEP; barriers to PrEP uptake included concerns about paying for PrEP and talking to one’s provider about sexual behaviors [23]. PrEP-related stigma has also been found to be a significant barrier to PrEP use among YMSM, including negative stereotypes about PrEP users [24-26]. Concerns about needing to use PrEP daily and possible side effects have also been found to serve as barriers to PrEP uptake. Additional factors inhibit transgender and nonbinary people from accessing PrEP [27]. In focus groups conducted with transgender men in San Francisco, barriers around lack of PrEP knowledge, financial concerns, finding a transcompetent doctor, and discussing sexual matters with providers emerged as key themes, mirroring barriers faced by young sexual minority cisgender men [28].

Patient activation, having the knowledge, skills, and self-efficacy to manage one’s health, is considered to be an important indicator of the willingness and ability to manage one’s own health and care autonomously [29,30]. Recent studies have found patient activation can significantly increase adherence and improve health-related outcomes [31-35]. Numerous studies have demonstrated that patients who are more activated in their care are also more likely to enact preventive behaviors, such as having regular checkups, screenings, and immunizations. Furthermore, research demonstrates that training individuals on how to ask questions and giving them the support to do so increases their participation in their own care and increases their activation levels [36]. A patient activation intervention may be ideally suited for use with SGMY, particularly if the goal is to activate them to become more engaged in their health, their use of health care and HIV prevention services, and PrEP uptake.

Patient navigation could also play an important role in helping SGMY access PrEP and PrEP care. Navigators help guide patients through the health care system and help patients set up medical appointments and get financial, legal, and social support. They may also help patients communicate with their health care providers. Often, patient navigators provide emotional support and help individuals access services to address their basic needs (eg, residential stability, food security, transportation, and language interpretation services) [37,38]. Innovative approaches to PrEP navigation, such as digital delivery using a mobile health (mHealth) app or digital platform, could help youth access care. Although numerous mHealth platforms now exist or are being developed, few are customized for inclusive use by SGMY or have been designed specifically for PrEP uptake. The PrEPresent app adapts an existing mHealth platform, developed by and for SGMY, and uses it to facilitate the delivery of PrEP navigation, increase knowledge about HIV and PrEP, increase intention to use PrEP, reduce PrEP stigma, and increase patient activation.

### Theoretical Framework for Intervention

PrEPresent is a PrEP navigation, activation, and support intervention that builds upon the patient activation theory, which is rooted in concepts of self-efficacy and locus of control [39-41], and in the transtheoretical model of change [42]. This model refers to “the individual’s knowledge, skills, and confidence in managing [their] own health and care” [43]. Patient activation theory describes an incremental process that patients undergo when becoming protagonists of their care management [44]. Building on this theory, we developed an intervention that is specifically targeted to increase PrEP uptake. Through both the mHealth platform itself and through interactions with PrEP navigators, participants are provided with developmentally appropriate information to (1) increase their self-awareness of their risk for HIV, (2) increase their awareness and knowledge about PrEP, and (3) address PrEP misperceptions and stigma. PrEP navigators also activate participants by increasing their PrEP self-efficacy and helping them develop strategies to address structural barriers related to PrEP uptake, such as obtaining insurance, finding an LGBTQ- and youth-friendly PrEP provider, obtaining transportation, finding a convenient pharmacy, obtaining and refilling PrEP prescriptions, paying for PrEP, developing a routine and reminders for taking a daily pill, and scheduling follow-up clinic visits.

### Aims and Objectives

This study aims to (1) conduct formative research to inform the development of PrEPresent using qualitative data from key informant interviews involving PrEP care providers and navigators and working groups with SGMY, (2) complete 2 rounds of usability testing of PrEPresent with 8-10 SGMY assessing both the intervention content and mHealth delivery platform to ensure features are usable and content is understood, and (3) evaluate the feasibility and acceptability of PrEPresent within a racially and ethnically diverse sample of 150 SGMY aged 16-26 years living in the Los Angeles area.

## Methods

### Ethics Approval

The research and ethics described in this study have been reviewed and approved by the institutional review board of Children’s Hospital Los Angeles (CHLA-20-00596). A certificate of confidentiality was obtained from the National Institutes of Health, and a waiver of parental consent has been obtained for participants aged 18 years or younger. This study is registered at ClinicalTrials.gov (NCT05281393).

### Phase 1: Formative Research

To inform the development of PrEPresent, we will conduct 2 sets of formative research activities (phase 1A and phase 1B). During phase 1A, we will conduct qualitative interviews lasting 60-minutes with 15 PrEP providers and PrEP navigators serving SGMY. Participants will receive US $35 to complete an interview and will be employed at health care or community-based organizations located in Los Angeles County, at least 18 years old, and of all genders. During phase 1B, we will form 2 working groups, each with 6-10 racially and ethnically diverse SGMY. Participants will meet via chat or videoconference on a weekly basis for a total of 4 weeks and each session will last 2 hours. Participants will be compensated US $50 for each session they attend and will receive an additional US $50 for active participation across the groups. Active participation includes attendance, contributing to session discussions, and participating in session activities. Participants will be (1) aged between 16 and 26 years; (2) cisgender male, transgender people, gender nonconforming, or identify differently from the gender picked for them at birth; (3) gay, bisexual, or some other same-sex identity, or report having had sex with anyone with a penis during the previous 12 months; (4) White or Caucasian, Black or African American, Latinx, Asian-Pacific Islander, Indigenous, Native American or multiracial; (5) living in the Los Angeles metro area; (6) have daily access to an iOS or Android smartphone or tablet with internet access; and (7) know their HIV status and be HIV-negative at time of enrollment.

We will engage participants in guided discussions on a variety of topics, including (1) general discussion around health and where participants access health information; (2) experiences discussing sexual health, sexual identity, sexuality, and gender with health care providers; (3) knowledge, attitudes, and beliefs about PrEP; (4) a PrEP journey activity (ie, use of a digital board game activity to facilitate discussion about the steps young LGBTQ people need to get and stay on PrEP and to develop possible app features and resources that will facilitate this); (5) how participants use mobile phone apps in their daily lives; (6) an app tracking activity (ie, each participant downloads an activity tracking app and reports back on what they like and dislike about the app, and how it could be improved); and (7) specific app features, such as avatars, badges, and discussion forums.

### Phase 2: Usability Testing

Following initial development of the PrEPresent digital platform, we will conduct usability testing with 8-10 racially and ethnically diverse SGMY. Study participants will participate in 1 digital focus group to download and review the app, 1 digital (chat or videoconference) session with our live PrEP Navigator (the “PrEPresentative”), and a one-on-one exit interview to provide feedback on the app. Participants will also be asked to complete pre- and posttest surveys to assess app experience and general usability. Participants will be compensated US $40 for each digital session they attend.

During the digital focus group, which will last 60-90 minutes, we will prompt participants for feedback in the following categories: understanding of features, overall impressions of the relevance of the intervention, and additional content or features that might be added to increase their likelihood of use. Then, participants will be instructed to use the app over a 2-week period and to schedule a 30-minute meeting with the PrEPresentative. PrEPresentative sessions will occur via chat or videoconference and will use the app’s scheduling and meeting features. These sessions will assess the feasibility of the newly developed features and provide an opportunity to gather feedback about the PrEPresentative role. The one-on-one exit interview will occur approximately 3 weeks after the digital focus group. This will allow participants time to use the app, generate feedback, and meet with the PrEPresentative. The interview, which will last 30-45 minutes, will include questions regarding overall intervention satisfaction, technical functionality and performance, and specific questions about each feature available in the app (medication tracker, resources, goal setting, forums, PrEPresentative, activities, badges, and avatars). Pre- and posttest surveys will be administered before downloading the app and after the 2-week testing period (but before the exit interviews to reduce response bias). Participants will meet the same eligibility criteria as outlined for phase 1B of the study (see above).

### Phase 3: Pilot Randomized Controlled Trial

#### Design

**Figure 1:**
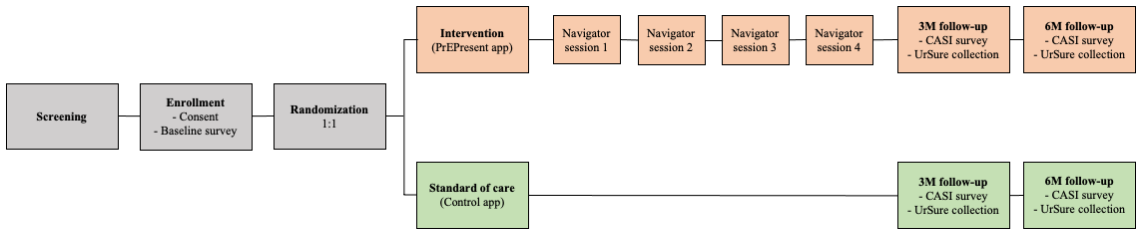
PrEPresent pilot randomized controlled trial study schema. 3M: 3 months; 6M: 6 months; CASI: computer-assisted self-interview.

The pilot randomized controlled trial (RCT) to evaluate intervention feasibility and acceptability will enroll up to 150 SGMY living in Los Angeles County, California, who will be randomized 1:1 to either the intervention (PrEPresent app) or control (information-only app) arms. The active intervention period is from 0 to 3 months and participants will complete study visits at baseline, 3, and 6 months (see Figure 1).

#### Participants

Participants will be (1) aged 16-26 years; (2) cisgender male, transgender people, gender nonconforming, or identify differently from their gender picked for them at birth; (3) gay, bisexual, or some other same-sex identity, or report having had sex with anyone with a penis during the previous 12 months; (4) White or Caucasian, Black or African American, Hispanic or Latino or Latinx, or multiracial with one of these identities; (5) living in the Los Angeles metro area; (6) have daily access to an iOS or Android smartphone or tablet with internet access; (7) report having insertive or receptive sex in the previous 6 months or report a positive STI result in the previous 6 months; (8) not currently on PrEP and have no plan to start or restart PrEP in the following 7 days; and (9) not currently enrolled in another HIV prevention study.

Prospective participants will be considered ineligible if they are non-English speaking, living with HIV, or if they participated in phase 1 or phase 2 study activities.

#### Recruitment

Individuals will be recruited from multiple in-person and digital sources. First, study staff will contact potentially eligible youth from 2 existing longitudinal cohorts (Healthy Young Men’s Cohort and the Transgender Youth of Color Cohort Study) who consented to being contacted about future research opportunities to assess their interest in and eligibility for PrEPresent. Staff will also use venue-based methods (eg, tabling events, referral from health or social service agencies) via flyers. Digital recruitment will include targeted advertisements on popular social media sites (eg, Facebook and Instagram). Potential participants recruited in any of the aforementioned ways will be directed to the study screening survey hosted on Qualtrics (Qualtrics Inc), a Health Insurance Portability and Accountability Act (HIPAA)–compliant digital software program. After reviewing a brief description of the study and providing consent to be screened, potential participants will complete the brief screening survey and provide contact information for follow-up use. Eligible SGMY will be invited to an initial in-person or digital visit in which staff will describe the study, confirm eligibility, obtain informed consent, sign a HIPAA release, and complete a baseline assessment. Youth who self-report living with HIV while reviewing eligibility criteria will be linked to care.

#### Randomization

Participants who meet eligibility criteria, consent to participation in the study, and complete the baseline survey will be randomized in a 1:1 ratio to the control or intervention arm using a prespecified randomization sequence that is reviewed by the study statistician. Randomization will not be stratified.

#### Intervention Arm

Participants assigned to the intervention will be asked to download the PrEPresent app and will be given a guided tour of the app and its main features (Table 1). Participants will be encouraged to schedule their first navigation session with the PrEPresentative and to use the app’s other features on a regular basis.

The PrEPresent platform will include live chat topics for the PrEP navigator, a repository of motivational text messages covering a range of identified barriers and facilitators to PrEP uptake, a curriculum for the PrEP navigator’s videoconferencing sessions, and a library of media and informational content to address identified gaps in PrEP knowledge, strategies, and skills. The app also includes an anonymous forum to facilitate peer-to-peer exchange, interactive activities, and a direct portal to engage in one-on-one interactions with the PrEPresentative either through text or video.

Participants randomized to the intervention arm will receive up to 4 sessions with the PrEPresentative over the 3-month intervention period (Table 2). Sessions will occur approximately 1 month apart, with session 1 beginning from the date of enrollment. Sessions will be scheduled via the PrEPresent app and can be scheduled, based on participant choice, via one of three ways: (1) text via app, (2) phone call, and (3) HIPAA-compliant videoconferencing (WebEx). During these sessions, the PrEPresentative will begin developing a tailored plan to link participants to PrEP care screening, HIV or STI testing, or prescriptions for PrEP. The PrEPresentative will also work with participants to increase patient activation (self-advocacy, self-efficacy, and motivation), help them identify a pharmacy to fill prescriptions, if necessary, help them pay for prescriptions (eg, insurance enrollment), and help develop practical, individualized behavioral strategies to support adherence. The PrEPresentative will check in via chat or videoconference at least weekly with participants to (1) ensure their needs continue to be met, and (2) provide reminders and prompts to ensure adherence to PrEP. The PrEPresentative will also use the mHealth platform to deliver motivational text messages, monitor PrEP follow-up visits, and monitor prescription refills. Participants can respond to these messages or engage in text messaging via the intervention app using the secure 2-way communication portal.

**Table 1:** Overview of PrEPresent app intervention features.

App feature	Description
Health tracker	Medication and habit tracking allows users to visualize patterns in their adherence, self-monitor, and receive feedback for improvement.
Forum	Users can participate in forum discussions and polls to foster community support and peer-to-peer sharing within the app.
Resources	Provides educational content across a range of health topics.
Activities	Supports app engagement and behavior change through information and skill-building.
Goals	Pushes tailored content through a curriculum that keeps users on target with health and wellness goals. Provides milestones, tasks, tips, connections, and journal opportunities related to participant’s goals.
Appointments	Connects users to a PrEP navigator to support them along their PrEP journey. Users can request times from available time slots, as well as a preferred method of contact (phone, text, or video).
Ask the expert	Subject matter experts answer anonymously submitted health and wellness questions from users. Questions and answers are visible to all participants to promote shared knowledge and connection to resources.
Avatar customization	Supports ongoing engagement by unlocking new accessories the more users engage with the app.
Care locator	Directory of local services specific to their need. Covers physical health, mental health, legal support, housing resources, and more.
Badges	Users can earn badges for actions taken within the app. Rewards engagement and participation in the study.

**Table 2:** Overview of PrEPresentative sessions.

Session	Time point	Delivery window	Topics
1	BL^a^	Days 0-14	Building rapport, introduction to intervention, overview of PrEPresentative role, PrEP^b^ motivation, and needs assessment.
2	1 month	Days 15-44	Developing tailored linkage to PrEP plans, adherence strategies (as needed), and supporting ongoing patient activation.
3	2 months	Days 45-74	Reviewing progress, follow-up and adjustments to tailored linkage to PrEP plans, adherence strategies (as needed), and supporting ongoing patient activation.
4	3 months	Days 75-104	Reviewing progress, intervention off-boarding (transition), reinforcement of adherence strategies (as needed), and maintenance of patient activation.
Check-ins	Weekly	Day determined at initial visit	Ensure needs continue to be met, provide reminders and prompts for linkage to PrEP care, medication refills, and adherence.

^a^BL: baseline.

^b^PrEP: pre-exposure prophylaxis.

#### Standard of Care Arm

Participants randomized to the standard of care arm will be given access to an information-only version of the PrEPresent app that only includes the library of media and informational resources regarding PrEP. These participants will not have access to the PrEPresentative sessions or other enhanced features of the app. Standard of care participants will be encouraged to use the control app on a regular basis.

#### Follow-Up Visits and Retention

At 3 and 6 months after enrollment, participants will be asked to complete a survey assessment and UrSure (UrSure Inc) at-home test kit to measure PrEP uptake. Study staff will contact participants prior to their target date using the participant’s preferred contact method indicated at baseline. Additional reminders will be provided via email, text, and phone call on and after the target date as needed to ensure completion of study visit procedures. Staff may leave messages (voicemails) for participants who consented to this at baseline.

#### Incentives

Participants will be compensated for completion of surveys at baseline and 3 and 6 months (US $50 each) and urine specimens at 3 and 6 months (US $20 each). If participants successfully complete all survey assessments and specimen collections, they will receive an additional US $50 bonus. Control arm participants can receive up to US $240, while intervention participants could receive up to US $280, as they will be asked to complete a brief satisfaction survey after each of their 4 PrEPresentative sessions (US $10 each).

#### Data Collection

At the time of enrollment, baseline surveys will be administered, while follow-up surveys will be administered 3- and 6-months postenrollment. Urine specimen results will be collected at 3- and 6-months postenrollment.

#### Primary Outcome Measures

We will assess the feasibility and acceptability of the patient navigation and mHealth platform aspects of the PrEPresent intervention. We will define patient navigation feasibility as at least half of intervention participants attending on average at least 2 sessions over the 3-month intervention period and mHealth platform feasibility as at least half of intervention participants using the platform on average at least 2 times per week over the 3-month intervention period. Patient navigation acceptability will be defined as a mean postnavigation satisfaction survey score of 4 or higher and mHealth platform acceptability as a score of greater than 70 using the 10-item System Usability Scale [45,46].

#### Secondary Outcome Measures

Preliminary efficacy of the PrEPresent intervention will include PrEP uptake measured using self-report PrEP use scales at the 3- and 6-month timepoints. Additionally, biological specimens using the UrSure point-of-care rapid urine test of tenofovir will be used. Potential mediators and moderators of the PrEPresent intervention, including PrEP self-efficacy, PrEP stigma, barriers to PrEP use, and patient activation will be assessed via 3- and 6-month surveys.

#### Statistical Analysis

Univariate statistics (eg, SD, range, and value) will be used to describe the study sample as well as values of the trial measures detailed above. Bivariate analyses will examine relationships among variables of interest. We will also produce summary scores and scales for constructs of interest (eg, System Usability Scale). Scale scores will be created as means or sums of unweighted composite scores depending on original item types.

To assess intervention feasibility and acceptability, we will summarize related data as means and proportions, with the 95% CI, by assessment period. Pre- to postintervention changes will be evaluated with generalized linear mixed models; a subject-level random intercept will be specified. Estimates of changes at each postintervention assessment will be calculated, along with the 95% CI.

We will follow steps for mediation analysis using nonparametric bootstrapping to assess indirect (mediated) effects and determine the CI [47,48]. Both the direct (unmediated) and indirect (mediated) effects of the intervention on each outcome of interest will be estimated and tested. Moderators (eg, racial and ethnic group) will be evaluated in these models, estimating and testing for differences in intervention effects across groups or other specified subgroups.

## Results

Phase 1 (formative work) began January 2021 and was completed April 2021. Usability testing began November 2021 and was completed December 2021. Institutional review board approval for the phase 3 (the pilot RCT) was granted in May 2022 and data collection began the same month. As of June 2023, 148 participants have been enrolled into the PrEPresent pilot RCT. Enrollment is expected to be completed in July 2023, with final results anticipated in December 2023.

## Discussion

### Overview

PrEPresent is a mHealth intervention that will address evidence-based barriers to engagement in PrEP care for SGMY in Los Angeles, California. The intervention is innovative in that its eligibility criteria are not strictly based on gender but prioritize populations engaging in sexual behaviors that qualify individuals for PrEP usage per CDC guidelines. PrEPresent leads with a patient activation strategy to encourage self-advocacy, self-efficacy, and motivation in accessing care within the HIV care continuum, particularly within sexual and gender minority populations who face medical stigma and structural disadvantages to engagement in affirming health care. The PrEPresentative, or patient navigator, will also use motivational interviewing techniques to address additional barriers such as HIV stigma, sexual wellness education, insurance and provider navigation, transportation, and housing difficulties.

### Limitations

During the creation of the PrEPresent intervention, care was taken to conduct formative research with PrEP providers and navigators in the Los Angeles areas to understand barriers and activators to uptake within a local landscape. This research is limited in its generalizability due to its centralization within a major metropolitan city and due to the location-specific resources available surrounding HIV-related care. Both California as a state and Los Angeles as a county offer universal health care coverage and have prioritized PrEP uptake and access within their HIV prevention efforts.

Participant eligibility criteria for the project included meeting the CDC qualifications for PrEP prescription. It is possible that fluctuations in participant sexual activity due to different factors may impact their willingness to uptake PrEP. A limitation of the current protocol is that PrEP uptake is only measured over a period of 6 months. Additional education and guidance throughout the intervention period may have longer-reaching impacts on PrEP willingness and use beyond this timeline.

Additionally, while efforts were made to include youth in formative research with expansive genders, ethnic identities, and experiences to inform tailored, affirming, and engaging app content, there may be population-specific areas that were not addressed among the resources available within the app.

### Conclusions

The PrEPresent intervention aims bridge the gaps in PrEP eligibility and PrEP uptake among racially and ethnically diverse SGMY. By facilitating the delivery of PrEP navigation and focusing on improving patient activation, the PrEPresent intervention has the potential to positively impact the PrEP uptake cascade within the HIV care continuum as well as serve as a model for the tailoring of PrEP interventions based on behavior-based qualifications for PrEP instead of generalized gender-based eligibility.

## Appendix

Multimedia Appendix 1 Peer-review report from HIBI, HIV/AIDS Intra- and Inter-personal Determinants and Behavioral Interventions Study Section (USA).

## Abbreviations

CDC: US Centers for Disease Control and Prevention
HIPAA: Health Insurance Portability and Accountability Act
LGBTQ: lesbian, gay, bisexual, transgender, and queer
mHealth: mobile health
PrEP: pre-exposure prophylaxis
RCT: randomized controlled trial
SGMY: sexual and gender minority youth
STI: sexually transmitted infection
YMSM: young men who have sex with men

## Data Availability

The data sets generated and analyzed during this study will be available from the corresponding author upon reasonable request.
