# Supplementary material for: Evaluating the Feasibility and Acceptability of a Digital Pre-Exposure Prophylaxis Navigation and Activation Intervention for Racially and Ethnically Diverse Sexual and Gender Minority Youth (PrEPresent): Protocol for a Pilot Randomized Controlled Trial
Source: JMIR Res Protoc. 2023 Sep 29;12:e50866. doi: 10.2196/50866 (PMC10576232; doi:10.2196/50866)
Supplement: Multimedia Appendix 1 [file resprot_v12i1e50866_app1.pdf]

SUMMARY STATEMENT
(Privileged Communication)

PROGRAM CONTACT:
Richard Jenkins
301-443-6504
jenkinsri@mail.nih.gov

Release Date: **12/13/2018**
Revised Date:

Application Number: **1 R34 DA046361-01A1**

Principal Investigator

KIPKE, MICHELE D.

Applicant Organization: CHILDREN'S HOSPITAL OF LOS ANGELES

Review Group: **HIBI**
HIV/AIDS Intra- and Inter-personal Determinants and Behavioral Interventions Study Section

Meeting Date: **11/15/2018**
Council: **JAN 2019**
Requested Start: **04/01/2019**
RFA/PA: **PA18-780**
PCC: **CV/RJP**

Project Title: **PrEP2Prevent: An Online PrEP Navigation and Activation Intervention for YMSM**

SRG Action: **++**
Next Steps: **Visit https://grants.nih.gov/grants/next_steps.htm**
Human Subjects: 30-Human subjects involved - Certified, no SRG concerns
Animal Subjects: 10-No live vertebrate animals involved for competing appl.

Project Year	Direct Costs Requested
1	200,000
2	200,000
3	50,000
TOTAL	450,000

++NOTE TO APPLICANT: Members of the Scientific Review Group (SRG) were asked to identify those applications with the highest scientific merit, generally the top half. Written comments, criterion scores, and preliminary impact scores were submitted by the assigned reviewers prior to the SRG meeting. At the meeting, the more meritorious applications were discussed and given final impact scores; by concurrence of the full SRG, the remaining applications, including this application, were not discussed or scored. The reviewers' comments (largely unedited by NIH staff) and criterion scores for this application are provided below. Because applications deemed by the SRG to have the highest scientific merit generally are considered for funding first, it is highly unlikely that an application with an ND recommendation will be funded. Each applicant should read the written critiques carefully and, if there are questions about the review or future options for the project, discuss them with the Program Contact listed above.

1R34DA046361-01A1 Kipke, Michele

DESCRIPTION (provided by applicant): This is a revised (A1) application entitled, PrEP2Prevent: An Online PrEP Navigation and Activation Intervention for YMSM, in which we propose to develop and pilot test a theoretically-driven, developmentally appropriate, and culturally relevant intervention for young men who have sex with men (YMSM). Our preliminary findings from research conducted with a cohort of at-risk YMSM suggests that an effective intervention to increase PrEP uptake and adherence should include three components: 1) inclusion of PrEP and other service navigation; 2) provision of skills to address patient activation to increase self-management in PrEP care; and 3) be delivered using mobile health (mHealth) technology. PrEP2Prevent aims to address anticipated or experienced barriers along the PrEP care continuum, to advance YMSM from PrEP awareness, to linkage to PrEP care and PrEP uptake, adherence, and ultimately persistence in PrEP care. PrEP2Prevent will address these barriers through the use of: 1) service navigation to address YMSM's unmet service needs, thus increasing their "readiness" for engagement in the PrEP care continuum, and 2) patient activation/self-management skills development to increase knowledge, motivation, skills and confidence for youth to manage their own health (e.g., through self-advocacy, self-efficacy, practical strategies). The multi-component intervention, delivered in-person and virtually during telehealth visits (both text and live video chat), builds off our teams' preliminary evidence with this population and our extensive experience delivering mHealth interventions. The specific aims are to: 1) conduct formative research to inform the development of the PrEP2Prevent intervention; 2) develop and conduct usability testing of the mHealth platform for intervention delivery; and 3) evaluate the feasibility, acceptability and preliminary efficacy of PrEP2Prevent to increase PrEP uptake and adherence with a racially/ethnically diverse sample of YMSM, ages 18-24 years. Surveys conducted at baseline, 3- (intervention completion) and 6-months, intervention process data as well as paradata on mHealth platform use will inform our primary feasibility and acceptability outcomes. Objective measures of PrEP use/adherence including verified service use, prescription refill data and biomarkers of adherence will be collected. Potential mediators (PrEP knowledge, intentions and self-efficacy, patient activation/self-management), and moderators (age, substance use, depression, sexual risk) of intervention efficacy will be assessed to inform intervention refinements and provide estimates of possible intervention effect sizes (group means, SDs) for a future randomized clinical trial. The proposed research, to develop a theory-driven, youth-friendly, culturally relevant PrEP navigation intervention focused on PrEP uptake and adherence among YMSM using a novel approach – patient activation – and an mHealth interface is highly significant, innovative and timely.

PUBLIC HEALTH RELEVANCE: Pre-exposure prophylaxis (PrEP) offers an opportunity to change the course of the HIV epidemic but the populations most affected by the epidemic, especially young men who have sex with men, experience multiple individual, social and structural factors that have limited their access to and initiation of PrEP. The proposed study will develop, implement and evaluate a theoretically-driven intervention to address these barriers and promote PrEP uptake and adherence using an existing mHealth platform. This new intervention, called PrEP2Prevent, will provide PrEP navigation and support to help youth access services to address their basic needs (e.g., food, housing, safety) and engage youth in each step of the PrEP care continuum -- i.e., build PrEP knowledge, motivation, uptake, adherence and retention.

CRITIQUE 1

Significance: 2
Investigator(s): 1
Innovation: 2
Approach: 4
Environment: 1

Overall Impact: This is a revised application to fund an R34 to develop and pilot a three component intervention to support uptake and adherence to PrEP among white, Black and Latino, young men who have sex with men (YMSM). The proposal will design an intervention with: 1) client-centered PrEP navigation, 2) patient activation; and 3) an mhealth platform to deliver intervention content. The final package, PrEP2Prevent, will be developed using formative research and work groups with young people; it will be piloted with 75 participants with three and six-month follow-up conducted to assess the following outcomes: PrEP use (via verified service use and prescription refill data) and adherence (using a biological outcome). Mediators will also be assessed. The results will inform powering and conduct of a full RCT. The proposal seeks to evaluate a combination of approaches that have strong potential for impact on the uptake of and adherence to PrEP. The focus on patient activation is novel and in this revised application well-justified. The study design is sound with a clearly laid out plan for achieving the study aims, which include conducting formative research, developing the intervention package through community-engaged methods, manualizing it and piloting the resultant program. The project is highly significant as well in that it focuses on a subpopulation in need of sustained and focused support in order to increase uptake of PrEP and reduce acquisition of HIV. The approach proposed is well-described integrating key intervention design components and a vigorous assessment of the feasibility and acceptability of the combination intervention. Overall the study investigators have addressed numerous critiques and suggestions of the previous review, although not all. The scientific premise is stronger, with the addition of background on the efficacy of patient activation and self-management on various health outcomes. There continue to be some minor weaknesses that reduce overall enthusiasm for the project. The theoretical mechanisms of action of patient activation have been better described, but the proposal is still light on how this will be applied in the intervention content. How the intervention addresses uptake is still not well described, although the significance makes clear that a major gap remains between PrEP awareness and uptake; the focus of the intervention still appears to be on youth who are interested in or willing to take up PrEP. The description of how patient activation will locate and activate PrEP aware and eligible, but not interested YMSM is not addressed. The intervention still appears very much to be an uptake (among interested individuals) and adherence intervention. Nonetheless, if successfully executed the study will pave the way for a large clinical trial of the multicomponent intervention for high-risk, Black and Latino, urban, YMSM, which is significant and could have high impact on the field and the epidemic.

1. Significance:

Strengths

- The subpopulations of focus are at very high risk for HIV acquisition.
- PrEP access, uptake and adherence is suboptimal for Black and Latino YMSM, although white YMSM are now included in this revised application.
- The revised application explains why transgender individuals are not included.
- PrEP is effective and barriers to uptake and adherence must be identified and addressed through in order to reach national HIV prevention goals
- The successful development of the intervention would offer an important model for US cities and YMSM.

Weaknesses

- The role of PrEP stigma is not well described.
- A major gap in the PrEP cascade is between eligibility and interest and intention to access. The data presented suggests that good portions of YMSM are aware of PrEP, but are not using it. Driving demand for PrEP by raising risk perception, addressing stigma and discrimination may

be where the work is needed, and not necessarily increasing access among those who seek PrEP.

- Self-management and patient activation have been used in conditions where there is a diagnosis; needing PrEP is not exactly the same.

2. Investigator(s):

Strengths

- PI is a senior leader in the HIV prevention field and experienced investigator with a strong track record of directing interdisciplinary research teams, NIH funding, complex research studies and reporting results in peer-reviewed publications.
- Highly experienced, interdisciplinary investigative team brings extensive and relevant expertise to project (e.g., population health, medical care, intervention development and testing, mhealth and technology science research, etc.)
- Appropriate balance of experienced senior investigators in required areas of expertise, which span various areas.
- Revised application adds significant strength in intervention design, with the addition of Bauermeister adding to the team considerably.
- It also adds new technology firms with considerable experience in mhealth and app development
- High level of support from Co-Investigators and collaborating clinical and community-based sites as demonstrated in letters of support

Weaknesses

- None Noted

3. Innovation:

Strengths

- Integrating mhealth with patient navigation and patient activation is innovative.
- Addressing multiple stages of the PrEP continuum is innovative

Weaknesses

- Patient navigation is not particularly innovative

4. Approach:

Strengths

- Although there are a lot of moving parts in this proposal, they are linked together in a way that makes sense and one builds on the next. The visual of the roadmap is helpful in indicating the process and structure of the work.
- The number of groups and group members of the “working groups” is now clear and seems adequate to the task.
- Again, how the formative research will clarify how patient activation “works” in the navigation and mhealth components is just not that well specified.
- The expansion of the working groups significantly increases confidence that the intervention design will be grounded in the real lives of the focal populations

- Stigma is mentioned and how the intervention might address it is described, albeit briefly.

Weaknesses

- The navigation component will address concrete and material needs, but the description of how this will happen is not very detailed; referrals will be made, but will the navigators be trained in case management and have the experience needed to do this effectively?
- PrEP stigma is not assessed in the pilot evaluation measures.
- The proposal indicates that it will develop a culturally relevant intervention, but there is little in the description of the formative research that addresses culture.

5. Environment:

Strengths

- All sites involved have excellent environments to conduct the research described.

Weaknesses

- None Noted

Study Timeline:

Strengths

- Detailed and clearly laid out for an ambitious project

Weaknesses

- None Noted

Protections for Human Subjects:

Acceptable Risks and/or Adequate Protections

- well described plan

Data and Safety Monitoring Plan (Applicable for Clinical Trials Only):

Acceptable

- well described

Inclusion of Women, Minorities and Children:

- Sex/Gender: Distribution justified scientifically
- Race/Ethnicity: Distribution justified scientifically
- For NIH-Defined Phase III trials, Plans for valid design and analysis: Not acceptable
- Inclusion/Exclusion of Children under 18: Including ages <18; justified scientifically

Vertebrate Animals:

Not Applicable (No Vertebrate Animals)

Biohazards:

Not Applicable (No Biohazards)

Resubmission:

- The applicants have addressed many of the concerns of the reviewers, although there remain some concerns.

Authentication of Key Biological and/or Chemical Resources:
Not Applicable (No Relevant Resources)

Budget and Period of Support:
Recommend as Requested

CRITIQUE 2

Significance: 4
Investigator(s): 3
Innovation: 2
Approach: 6
Environment: 4

Overall Impact: This is a revised application from Los Angeles Children's Hospital. The PI is an experienced researcher in HIV and youth. The proposed study will develop, implement and evaluate a theoretically-driven intervention to address these barriers and promote PrEP uptake and adherence using an existing mHealth platform. The proposed study has a number of strengths. It proposes an innovative approach to developing an intervention to improve the use of PrEP, and it has a with a talented team of investigators. There are also a number of weaknesses. It is not clear whether the project is planning to create a program for YMS overall, or three programs adapted for different ethnic groups. The general aim seems to be the former, but the methods seem oriented to the latter. A number of aspects of the approach are unclear or unwieldy, which seems likely to handicap the operation of the research. On balance, the research seems a fine idea but needs more clarity and focus.

1. Significance:

Strengths

- Young men who have sex with men (YMSM) are an important group in the HIV epidemic, as they continue to have high rates of new HIV infections and relatively low use of PrEP.
- If this project achieves its aims, it can develop a promising adaptation (or adaptions) of PrEP for YMSM.

Weaknesses

- The current application includes three target groups of YMSM African Americans, Hispanic Whites, and Non-Hispanic Whites - with what appears to be separate plans for both qualitative and quantitative analyses for each of them, without sufficiently specifying how (or if) the various results will be combined in the end. It is not clear if it is the goal to have a single PrEP, or three PrEP protocols designed for each of the groups.

2. Investigator(s):

Strengths

- There is a multidisciplinary team headed by an experienced research leader Dr. Kipke, who also serves as co-director of the Southern California CTSI.
- Co-investigators include adolescent medicine expert Marvin Belzer wo provides feedback ion PrEP guidelines and cultural anthropologist Katrina Kubicek, who verses the formative research Phase 1 of the study.
- Consultants include pharmacologist Angela Kashuba, whose lab analyzes dried blood spots for PrEP adherence, Joseph Baumeister, who consults on intervention development and evaluation, and Kate Muessig, a health behavior interventionist who will assist in the qualitative analyses.

Weaknesses

- It is difficulty to sort through the potential overlapping roles of the various leaders, consultants, subcontractors, and staff.

3. Innovation:

Strengths

- It is innovative to integrate mHealth services with patient navigation in the service of enrolling and helping YMSM to succeed in PrEP.
- The project will integrate a number of mHealth services in the PrEP2Prevent intervention.
- Using patient activation as a central concept seems to fit the YMSM population very well and is innovative.

Weaknesses

- None Noted

4. Approach:

Strengths

- The intervention, called PrEP2Prevent, will provide PrEP navigation and support to help youth access services to address their basic needs (e.g., food, housing, safety) and engage youth in each step of the PrEP care continuum.
- The PrEP2Prevent will include a major focus for patient navigation, which seems important for the young population. Another emphasis is emphasizing patient activation, which can help youth grow to represent their needs rather than relying on others to provide the impetus. A third emphasis is using technology support, which fits better with the young population than it would for an older clientele.

Weaknesses

- It is not clear what the primary and secondary dependent variables are. The distinction between feasibility and acceptability is not clearly stated, nor are the component measures clearly provided. “Feasibility” is defined as “at least 50% of individuals used the platform on average greater or equal to twice a week…”, but this is a narrow operational definition. There is no definition of “acceptability” provided.

- Many measures are indicated, but there is not a clear conceptualization or measurement model to guide what is needed.
- Expectations of the technological aspects may be unrealistic. For example, the CAB noted that low PrEP uptake may be related to patients needing to enter a clinic, and providing these services online would address this barrier. But online services may have the same problem with waiting lists, and at some point the client needs to be seen physically.
- Many aspects of the study seem unwieldy. For example, six working groups will be formed (3 ethnicity groups in PrEP or not in PrEP) to carry out some of the formative research, and this seems a substantial load: 6 groups x 4-6 sessions each, for 6-8 YMSM. It isn't clear how the results will be analyzed given the magnitude of the data, and it is not clear how the results will be synthesized across the groups.
- It would be helpful to include a section in the Approach recognizing limitations of the research, problems anticipated, and strategies to minimize problems. Such a section could better demonstrate awareness of potential drawback.

5. Environment:

Strengths

- Children's Hospital of Los Angeles (CHLA) is a strong home base for the study, and the PI has a key role there as Director of Research. Dr. Kipke is also the founder of CHLA's Community, Health Outcomes, and Intervention Research (CHOIR) Program.
- The Saban Research Institute is responsible for providing administrative support for research at CHLA, and all its PIs are members of the USC faculty.
- University of North Carolina provides support for Dr Hightow-Weidman and consultant Dr. Muessig, as well as analysis of laboratory tests under the supervision of Dr. Kashuba's laboratory.
- Two technology companies are also subcontractors, one will use focus group results to create a final mHealth design, and the other will use that design to create a desktop and/or mobile app for the YMSM clients as well as a research dashboard.

Weaknesses

- It is not very clear from the proposal what whether TSRI is a part of CHLA, vice-versa , or if they are independent of each other. Likewise what University of Southern California contributes to the study, if it does.
- While the team approach is useful, there are also many organizations and individuals to be coordinated in conducting the research.

Study Timeline:

Strengths

- Timeline is reasonable.

Weaknesses

- None Noted

Protections for Human Subjects:

Acceptable Risks and/or Adequate Protections

- Thoughtfully designed.

Data and Safety Monitoring Plan (Applicable for Clinical Trials Only):

Acceptable

Inclusion of Women, Minorities and Children:

- Sex/Gender: Distribution justified scientifically
- Race/Ethnicity: Distribution justified scientifically
- For NIH-Defined Phase III trials, Plans for valid design and analysis: Not acceptable
- Inclusion/Exclusion of Children under 18: Including ages <18; justified scientifically
- All men; justified scientifically. Race: Three groups will be included in approximately the same proportion, although the inclusion enrollment report is not very clear.

Vertebrate Animals:

Not Applicable (No Vertebrate Animals)

Biohazards:

Not Applicable (No Biohazards)

Resubmission:

- The prior review comments views praised the study's significance, research team, and innovation and commented that the work built on approaches that have strong potential for impact on the uptake and adherence of PrEP.
- But the reviewers were critical of many aspects including: 1) limiting participants to African Americans and Non-Hispanic whites, not including transgender participants, and not justifying why the study is restricted to men; 2) not adequately describing the theoretical mechanisms of action of patient activation or the contact delivered via Lifespeed; 3) "conducting a LOT" of formative research that is not well described; 4) not explaining how the intervention addresses stigma; 5) the cost of the intervention, particularly Lifespeed; 6) not clarifying what the study's primary and secondary variables are or the dependent variables, while including a large number of key study variables and not defining the key variables of "acceptability" or "feasibility"; and 8) not addressing cost measurement or tracking. The revision is responsive to some of the critiques, and it clearly indicates in the proposal in bold burgundy what changes were made. Many of the prior problems remain.

Authentication of Key Biological and/or Chemical Resources:

Acceptable

Budget and Period of Support:

Recommend as Requested

CRITIQUE 3

Significance: 2
Investigator(s): 1
Innovation: 5
Approach: 4
Environment: 2

Overall Impact: This revised R34 study by a PI from the Children's hospital of Los Angeles proposes an intervention to increase uptake of and adherence to PrEP among young men who have sex with men. As this group has some of the highest HIV incidence and prevalence in the US, it addresses a significant issue. The innovation is somewhat limited as the projects proposes to adapt a previously developed intervention (for African American MSM) to a new target population (young MSM). The main contribution of the project seems to be the direct targeting of patient activation. This is overall a strong application, with an appropriate responsiveness to the reviewer comments on the original submission, except for a key concept (patient engagement with technology) that is increasingly being recognized as a potential barrier for the implementation of mHealth interventions.

1. Significance:

Strengths

- Addresses a particularly vulnerable group
- Uses methods of implementation that are potentially scalable

Weaknesses

- None Noted

2. Investigator(s):

Strengths

- Team of investigators with extensive NIH funding experience and expertise in key areas for the application
- Appropriate effort levels of investigators

Weaknesses

- None Noted

3. Innovation:

Strengths

- Patient activation seems to be a novel construct for intervention

Weaknesses

- Adapts an intervention previously developed by the PI to a slightly altered population

4. Approach:

Strengths

- Explicitly theory-based intervention

Weaknesses

- Does not take potential obstacle of patient (dis)engagement into account
- No cut-offs for feasibility / acceptability thresholds given

5. Environment:

Strengths

- The academic and community-partner sites provide an excellent environment to carry out the study

Weaknesses

- None Noted

Study Timeline:

Strengths

- appropriate

Weaknesses

- None Noted

Protections for Human Subjects:

Acceptable Risks and/or Adequate Protections

Data and Safety Monitoring Plan (Applicable for Clinical Trials Only):

Acceptable

Inclusion of Women, Minorities and Children:

- Sex/Gender: Distribution justified scientifically
- Race/Ethnicity: Distribution justified scientifically
- For NIH-Defined Phase III trials, Plans for valid design and analysis: Not applicable
- Inclusion/Exclusion of Children under 18: Including ages <18; justified scientifically

Vertebrate Animals:

Not Applicable (No Vertebrate Animals)

Biohazards:

Not Applicable (No Biohazards)

Resubmission:

- largely responsive, yet the important potential problem of patient engagement pointed out in the original critique not addressed

Authentication of Key Biological and/or Chemical Resources:

Not Applicable (No Relevant Resources)

Budget and Period of Support:

Recommend as Requested

Footnotes for 1 R34 DA046361-01A1; PI Name: Kipke, Michele D.

NIH has modified its policy regarding the receipt of resubmissions (amended applications). See Guide Notice NOT-OD-14-074 at http://grants.nih.gov/grants/guide/notice-files/NOT-OD-14-074.html. The impact/priority score is calculated after discussion of an application by averaging the overall scores (1-9) given by all voting reviewers on the committee and multiplying by 10. The criterion scores are submitted prior to the meeting by the individual reviewers assigned to an application, and are not discussed specifically at the review meeting or calculated into the overall impact score. Some applications also receive a percentile ranking. For details on the review process, see http://grants.nih.gov/grants/peer_review_process.htm#scoring.

MEETING ROSTER

HIV/AIDS Intra- and Inter-personal Determinants and Behavioral Interventions Study Section
AIDS and Related Research Integrated Review Group
CENTER FOR SCIENTIFIC REVIEW
HIBI
11/15/2018 - 11/16/2018

Notice of NIH Policy to All Applicants: Meeting rosters are provided for information purposes only. Applicant investigators and institutional officials must not communicate directly with study section members about an application before or after the review. Failure to observe this policy will create a serious breach of integrity in the peer review process, and may lead to actions outlined in NOT-OD-14-073 at https://grants.nih.gov/grants/guide/notice-files/NOT-OD-14-073.html and NOT-OD-15-106 at https://grants.nih.gov/grants/guide/notice-files/NOT-OD-15-106.html, including removal of the application from immediate review.

CHAIRPERSON(S)

STEPHENSON, ROB B, PHD
PROFESSOR
DEPARTMENT OF HEALTH BEHAVIOR
AND BIOLOGICAL SCIENCES
SCHOOL OF NURSING
UNIVERSITY OF MICHIGAN
ANN ARBOR, MI 48109

MEMBERS

BUTLER, LISA MICHELLE, PHD *
ASSOCIATE RESEARCH PROFESSOR
INSTITUTE FOR COLLABORATION ON HEALTH,
INTERVENTION, AND POLICY
UNIVERSITY OF CONNECTICUT
STORRS, CT 06269

CHAMPION, JANE DIMMITT, DNP, PHD *
PROFESSOR
SCHOOL OF NURSING
UNIVERSITY OF TEXAS AT AUSTIN
AUSTIN, TX 78701

CHEN, XINGUANG, MD, PHD *
PROFESSOR
DEPARTMENT OF EPIDEMIOLOGY
COLLEGE OF PUBLIC HEALTH AND HEALTH PROFESSIONS
UNIVERSITY OF FLORIDA
GAINSVILLE, FL 32610

CORSI, KAREN F, SCD
ASSOCIATE PROFESSOR
DEPARTMENT OF PSYCHIATRY
SCHOOL OF MEDICINE
UNIVERSITY OF COLORADO, DENVER
DENVER, CO 80206

FRYE, VICTORIA, DRPH *
ASSOCIATE MEDICAL PROFESSOR
DEPARTMENT OF COMMUNITY HEALTH
AND SOCIAL MEDICINE
SCHOOL OF MEDICINE
CITY UNIVERSITY OF NEW YORK
NEW YORK, NY 10035

GWADZ, MARYA, PHD
SENIOR RESEARCH SCIENTIST AND DIRECTOR
TRANSDISCIPLINARY RESEARCH METHODS CORE
CENTER FOR DRUG USE AND HIV RESEARCH
RORY MEYERS COLLEGE OF NURSING
NEW YORK UNIVERSITY
NEW YORK, NY 10010

HANSEN, NATHAN B, PHD *
DEPARTMENT HEAD AND PROFESSOR
DEPARTMENT OF HEALTH PROMOTION AND BEHAVIOR
COLLEGE OF PUBLIC HEALTH
UNIVERSITY OF GEORGIA
ATHENS, GA 30602

HORVATH, KEITH JOSEPH, PHD
ASSOCIATE PROFESSOR
DIVISION OF EPIDEMIOLOGY AND COMMUNITY HEALTH
SCHOOL OF PUBLIC HEALTH
UNIVERSITY OF MINNESOTA
MINNEAPOLIS, MN 55454

KATZ, INGRID THERESA, MD *
ASSISTANT PROFESSOR
DEPARTMENT OF MEDICINE
BRIGHAM AND WOMEN'S HOSPITAL
BOSTON, MA 02115

KIENE, SUSAN MARIA, PHD *
PROFESSOR
DIVISION OF EPIDEMIOLOGY AND BIOSTATISTICS
SCHOOL OF PUBLIC HEALTH
SAN DIEGO STATE UNIVERSITY
SAN DIEGO, CA 92182

LECHUGA, JULIA, PHD *
ASSISTANT PROFESSOR
DEPARTMENT OF COUNSELING PSYCHOLOGY
COLLEGE OF EDUCATION AND HUMAN SERVICES
LEHIGH UNIVERSITY
BETHLEHEM, PA 18015

LINNEMAYR, SEBASTIAN, PHD
SENIOR ECONOMIST
RAND CORPORATION
SANTA MONICA, CA 90407

LIU, ALBERT YING-HWA, MD *
ASSISTANT PROFESSOR
SAN FRANCISCO DEPARTMENT OF PUBLIC HEALTH
DIRECTOR OF HIV PREVENTION INTERVENTION STUDIES
UNIVERSITY OF CALIFORNIA, SAN FRANCISCO
SAN FRANCISCO, CA 94102

LIU, HONGJIE, PHD *
PROFESSOR
DEPARTMENTS OF EPIDEMIOLOGY
AND BIOSTATISTICS
SCHOOL OF PUBLIC HEALTH
UNIVERSITY OF MARYLAND, COLLEGE PARK
COLLEGE PARK, MD 20742

MIMIAGA, MATTHEW JAMES, MPH, SCD
PROFESSOR
BEHAVIORAL AND SOCIAL HEALTH SCIENCES
AND EPIDEMIOLOGY
SCHOOL OF PUBLIC HEALTH
BROWN UNIVERSITY
PROVIDENCE, RI 02903

OWNBY, RAYMOND L, MD, PHD *
PROFESSOR AND CHAIR
DEPARTMENT OF PSYCHIATRY AND BEHAVIORAL MEDICINE
COLLEGE OF OSTEOPATHIC MEDICINE
NOVA SOUTHEASTERN UNIVERSITY
FORT LAUDERDALE, FL 33314

PATTERSON, THOMAS L, PHD *
PROFESSOR
DEPARTMENT OF PSYCHIATRY
UNIVERSITY OF CALIFORNIA, SAN DIEGO
LA JOLLA, CA 92093

RAMSEY, SUSAN E, PHD
ASSOCIATE PROFESSOR
DIVISION OF GENERAL INTERNAL MEDICINE
RHODE ISLAND HOSPITAL
BROWN UNIVERSITY
PROVIDENCE, RI 02903

RAO, DEEPA, PHD *
ASSOCIATE PROFESSOR
DEPARTMENT OF GLOBAL HEALTH
SCHOOL OF MEDICINE
UNIVERSITY OF WASHINGTON
SEATTLE, WA 98104

RHODES, SCOTT D, MPH, PHD
PROFESSOR
DEPARTMENT OF SOCIAL SCIENCES AND HEALTH POLICY
SCHOOL OF MEDICINE
WAKE FOREST UNIVERSITY
WINSTON-SALEM, NC 27157

SCHNALL, REBECCA, BSN, PHD, RN *
MARY DICKEY LINDSAY ASSOCIATE PROFESSOR
HEALTH PROMOTION AND DISEASE PREVENTION
SCHOOL OF NURSING
COLUMBIA UNIVERSITY
NEW YORK, NY 10032

SORENSEN, JAMES L, PHD
PROFESSOR
DEPARTMENT OF PSYCHIATRY
UNIVERSITY OF CALIFORNIA, SAN FRANCISCO
SAN FRANCISCO, CA 94110

SSEWAMALA, FRED M, PHD *
PROFESSOR
INSTITUTE FOR PUBLIC HEALTH
BROWN SCHOOL
WASHINGTON UNIVERSITY IN ST LOUIS
ST. LOUIS, MO 63130

STARKS, TYREL J, PHD *
ASSOCIATE PROFESSOR
DEPARTMENT OF PSYCHOLOGY
HUNTER COLLEGE
NEW YORK, NY 10065

STEKLER, JOANNE DONNA, MD *
ASSISTANT PROFESSOR
DIVISION OF ALLERGY AND INFECTIOUS DISEASES
DEPARTMENT OF MEDICINE
HARBORVIEW MEDICAL CENTER
UNIVERSITY OF WASHINGTON
SEATTLE, WA 98104

TOBIN, KARIN E, PHD
ASSOCIATE PROFESSOR
DEPARTMENT OF HEALTH, BEHAVIOR, AND SOCIETY
BLOOMBERG SCHOOL OF PUBLIC HEALTH
JOHNS HOPKINS UNIVERSITY
BALTIMORE, MD 21205

TURAN, JANET M, MPH, PHD
PROFESSOR
DEPARTMENT OF HEALTH CARE ORGANIZATION
AND POLICY
SCHOOL OF PUBLIC HEALTH
UNIVERSITY OF ALABAMA AT BIRMINGHAM
BIRMINGHAM, AL 35294

VREEMAN, RACHEL CHRISTINE, MD
ASSOCIATE PROFESSOR
DEPARTMENT OF PEDIATRICS
SCHOOL OF MEDICINE
INDIANA UNIVERSITY
INDIANAPOLIS, IN 46202

WALDROP-VALVERDE, DRENNA, PHD
ASSOCIATE PROFESSOR
DEPARTMENT OF NURSING
NELL HODGSON WOODRUFF SCHOOL OF NURSING
EMORY UNIVERSITY
ATLANTA, GA 30322

WILTON, LEO, PHD *
PROFESSOR
DEPARTMENT OF HUMAN DEVELOPMENT
COLLEGE OF COMMUNITY AND PUBLIC AFFAIRS
BINGHAMTON UNIVERSITY
BINGHAMTON, NY 13902

WINDSOR, LILIANE CAMBRAIA, PHD *
ASSOCIATE PROFESSOR
SCHOOL OF SOCIAL WORK
THE UNIVERSITY OF ILLINOIS AT URBANA-CHAMPAIGN
URBANA, IL 61801

SCIENTIFIC REVIEW OFFICER

RUBERT, MARK P, PHD
SCIENTIFIC REVIEW OFFICER
CENTER FOR SCIENTIFIC REVIEW
NATIONAL INSTITUTES OF HEALTH
BETHESDA, MD 20892

EXTRAMURAL SUPPORT ASSISTANT

STROTHERS, DIARA
EXTRAMURAL SUPPORT ASSISTANT
CENTER FOR SCIENTIFIC REVIEW
NATIONAL INSTITUTES OF HEALTH
BETHESDA, MD 20892

* Temporary Member. For grant applications, temporary members may participate in the entire meeting or may review only selected applications as needed.

Consultants are required to absent themselves from the room during the review of any application if their presence would constitute or appear to constitute a conflict of interest.
